# 

**Published:** 1843-03-18

**Authors:** 


CONTENTS.

Dr. Bailey's Case of Hepatic Colic, . 49

*Foreign Correspondence,*

Letter from Paris, . . . 50

Board of Naval Surgeons, . . . . 50

*Jefferson Medical College.*

Clinic of Professor Mütter.

False Ankylosis of the elbow-joint, &c. 51

*Bibliographical Notice.*

Valedictory Address delivered before the Graduates of Pennsylvania Medical College: Session of 1842–'3. By Robert M. Bird, M. D., Professor of Materia Medica, and Institutes of Medicine, . . . . . . . 54

Syphilitic Eruptions, . . . . 55

Green Cataract, . . . . . 55

New method of administering quinine. By Dr. Guastamacchia, . . . . 55

*Retrospect of the Medical Sciences.*

False Jalap, . . . . . . 56

Taliacotian operation. By J. Mason Warren, M. D., of Boston, . . 56

Observations on a particular form of encysted tumour, which occurs in the neck, but is not necessarily connected with the thyroid body. By Benjamin Phillips, F. R. S., &c. . . . . 57

English Physicians. By Prof. Marx, 58

Nervous Ophthalmia, . . . . 58

New mode of Extraction of Cataract. By Dr. Bonnet, of Lyons, . . 58

On the Chemical Composition of the Fluid of Ranula. By L. Gmelin, . 59

On the Fractures of the Lower End of the Radius. By M. Voillemier, . 59

Prevention of Lead Colic, . . . . 60

Robertson on the cause of Caries of the Teeth, . . . . . . . 60

Revival after Freezing, . . . . 60

